# Plasma lyso-phosphatidylcholine concentration is decreased in cancer patients with weight loss and activated inflammatory status

**DOI:** 10.1186/1476-511X-6-17

**Published:** 2007-07-10

**Authors:** Lenka A Taylor, Jann Arends, Arwen K Hodina, Clemens Unger, Ulrich Massing

**Affiliations:** 1 Tumor Biology Center, Dept. of Clinical Research, Breisacher Straße 117, D-79106 Freiburg, Germany

## Abstract

**Background:**

It has been observed that ras-transformed cell lines in culture have a higher phosphatidylcholine (PC) biosynthesis rate as well as higher PC-degradation rate (increased PC-turnover) than normal cells. In correspondence to these findings, the concentrations of the PC-degradation product lyso-phosphatidylcholine (LPC) in cancer patients were found to be decreased. Our objective was the systematic investigation of the relationship between LPC and inflammatory and nutritional parameters in cancer patients. Therefore, plasma LPC concentrations were assessed in 59 cancer patients and related to nutritional and inflammatory parameters. To determine LPC in blood plasma we developed and validated a HPTLC method.

**Results:**

Average plasma LPC concentration was 207 ± 59 μM which corresponds to the lower limit of the reported range in healthy subjects. No correlation between LPC and age, performance status, body mass index (BMI) or fat mass could be seen. However, LPC correlated inversely with plasma C-reactive protein (CRP) and whole blood hydrogen peroxides (HPO). Further, a negative correlation could be observed between LPC and whole body extra cellular fluid volume (ECF) as well as with relative change in body weight since cancer diagnosis.

**Conclusion:**

In conclusion, LPC concentrations were decreased in cancer patients. LPC plasma concentrations correlated with weight loss and inflammatory parameters and, therefore, might be a general indicator of severity of malignant disease.

## Background

Phosphatidylcholine (PC), a glycerophospholipid bearing a polar phosphocholine head group and two non-polar fatty acid hydrocarbon chains, represents the main membrane-forming phospholipid in mammalian cells. Removal of one of the fatty acids, enzymatically or by spontaneous hydrolysis, results in lyso-phosphatidylcholine (LPC). In contrast to PC, which is a membrane forming phospholipid, LPC exerts a lytic action on membranes [1]. In living cells, LPC usually results from membrane PC through the enzymatic action of a phospholipase A_2 _(PLA_2_), which cleaves the fatty acid from the 2-position of the glycerol backbone. In the blood, LPC is usually generated by PLA_2 _or by the lecithin-cholesterol-acyl-transferase (LCAT) from PC present in lipoproteins. The latter enzyme transfers a fatty acid from PC to cholesterol, resulting in a cholesterolester and LPC [2]. It has been reported that triglyceride lipases on endothelial cells are also able to generate LPC by cleaving the fatty acid ester bond in the 1 position of glycerophospholipids in lipoproteins [3]. Since LPC is membrane lytic, it is predominantly bound to albumin in the blood and the amount of free, monomolecular dissolved LPC which is in equilibrium with albumin-bound LPC – is very small. However, even though the equilibrium is shifted strongly towards albumin-bound LPC, LPC transfer into cells seems to be fast. It can be taken up by cells rapidly and LPC released into the blood from membrane PC is quickly bound to albumin. Thus, the albumin system keeps LPC concentrations in the blood below the lytic concentration but at the same time provides rapid supply if necessary.

The concentration of LPC in blood plasma of healthy persons usually ranges from 200 to 300 μM [4,5]. Our own unpublished observations confirmed this range. Sullentrop et al. as well as Kuliszkiewicz-Janus et al. have reported slightly higher ranges of LPC for healthy persons, varying between 300 and 400 μM [6,7].

It has been observed that ras-transformed cell lines have a higher PC-turnover and a higher consumption of LPC than normal cells [8]. Elevated pools of two PC breakdown products, phosphocholine and glycerophosphocholine, were observed in these experiments [9]. It has been reported that the ras oncogene product directly or indirectly causes an increased turnover of PC in mouse fibroblast cells [10]. Another study indicated that PC hydrolysis is a target of Ras during the transduction of growth factor-initiated mitogenic signals [11].

In correspondence to the finding that tumour cells consume more LPC than normal cells, patients with malignant diseases have been found to show changes of the usual plasma phospholipid pattern. The analysis of plasma lipids in a group of patients with different kinds of cancer revealed a general decrease of phospholipids [12]. A group of patients with leukemia, malignant lymphomas as well as gastrointestinal and renal tumours was found to have decreased LPC concentrations even at an early stage of disease compared to healthy persons [6]. In patients with acute leukemia significantly diminished plasma LPC concentrations normalised with treatment induced disease remission [7]. Similar results were observed in patients with renal cancer. In comparison with healthy volunteers, both male and female patients had decreased LPC concentrations in plasma which could be related to tumour status and metastasis [13].

Based on these findings, we postulated that decreased plasma LPC concentrations might be a general indicator for malignant disease and may even allow a predication of the state of disease. We, therefore, examined the LPC concentrations in a group of 59 cancer patients with different disease entities and varying stages of disease progression. We determined inflammatory parameters as well as the nutritional status, since severity of disease is generally associated with an activation of systemic inflammatory processes and a deterioration of nutritional status [14].

## Results and Discussion

### Quantification of LPC by HPTLC – Method validation (see Table 1)

**Table 1:** Summary of validation results

**analyte:**	lyso-phosphatidylcholine
**sample:**	75 μl human plasma
**selectivity:**	given
**calibration range:**	60 – 480 μM
**analytical function:**	quadratic equation (peak area)
**linearity:**	correlation coefficient r ≥ 0.991
**precision and accuracy**	
**calibration:**	inter day accuracy ± 6.7% and precision ± 5.5%
**QC samples precision:**	
**intra-assay:**	± 11%
**inter-assay:**	± 19%
**LOQ**	60 μM (inter day accuracy ± 4.7% and precision ± 5.1%)
**LOD**	< 40 μM
**Recovery from plasma**	79% ± 2%

The HPTLC is a well-known method in phospholipid analysis but has to our knowledge not yet been employed to quantify LPC in human blood plasma. The validation is therefore a necessary prerequisite for the clinical use of this method and allows comparison with measurement results obtained by other means, such as NMR or HPLC. The advantages of the HPTLC method are its comparable low cost and the possibility to analyse up to 12 samples in one run.

Selectivity, linearity, precision and accuracy as well as limit of quantification, recovery from lipid extraction and stability of analyte under various storage conditions were assessed.

The concentration of calibration standards ranged from 60 to 480 μM which were used on 6 different days independently to estimate intra- and interday precision and accuracy same as the QC samples 6 times per day to estimate precision. For QC samples pooled human plasma with an unknown amount of LPC was used.

### Stability of LPC in blood plasma

As there was nothing known about the stability of LPC in blood plasma under various handling and storing conditions, this part of the validation was a very important aspect.

The concentration of LPC in blood plasma increases rather quickly over time if stored at room temperature (+ 25°C). The net rise after 48 hours can be estimated at about 170% of the concentration at zero hours.

Storage of samples in the refrigerator (+ 7°C) does not affect the concentration of LPC in blood plasma. There is neither an increase nor decrease to be observed during 72 hours of examination.

Long term storage (6 months) at a temperature of -80°C does not affect the LPC concentration in plasma sample aliquots.

Three freeze and thaw cycles from -80°C to room temperature with immediate deep freezing after sample collection do not influence the amount of LPC in the plasma sample.

Stability of samples was assessed for the first time and the results show that it is possible to store samples by freezing over large time spans until accumulation of maximum number of samples for one run which makes the analysis more efficient than having to process samples straight away.

The validation of the HPTLC method clearly showed that it is suitable for sufficiently reproducible, precise and accurate analysis of LPC in human blood plasma.

## Patient study

### Patients characteristics (see Table 2)

**Table 2:** Patients' characteristics: summary of the examined parameters with analysis of distribution

**parameter**	**average**	**range**
**age (years)^1^**	60.6 ± 10	32 to 78
**performance status^1^**	1.5 ± 0.7	0 to 3
**BMI (kg/m^2^)**	25.6 ± 4.1	18 to 36
**ΔFD (% BW)**	-1.9 ± 11.8	-28.8 to + 23.3
**FM (% BW)**	23.7 ± 8.3	9.4 to 42.4
**HPO (U.FORT)^1^**	463 ± 150	156 to 938
**CRP (mg/dl)^1^**	1.7 ± 3	0.5 to 21.1
**ECF (%TBW)**	48.8 ± 4	41 to 60
**albumin (g/dl)^1^**	3.8 ± 0.6	1.7 to 5.7
**LPC (μM)**	207 ± 59	89 to 362

^1^non-normal distribution

Age, BMI and the degree of weight change differed considerably among the study subjects. No patient was severely underweight. The average weight change since tumour diagnosis was small, while some patients had lost a large fraction of their body weight and others had gained weight substantially. Also body composition differed remarkably among the patients and fractional fat mass ranged from 9 to 42%, the higher value corresponding to the upper limit of the normal range for women aged 55 to 64 years [15].

HPO are derived from physiologic lipids by oxidative damage and HPO blood concentrations are taken to be an adequate parameter to evaluate oxidative stress [16]. Oxidative damage marks high metabolic activity but also inflammatory processes. HPO concentrations detected in our patients in several cases exceeded the normal range published by an Italian group [17] considerably; this corresponds to our previous observations of increased HPO concentrations in cancer patients [18].

Average CRP concentrations were above the normal range (> 1 mg/dl) [19] and individual patients had excessively elevated concentrations indicating varying degrees of activated inflammatory status.

Albumin is considered to be a negative inflammatory marker. Average albumin concentrations were normal [20] but many patients had subnormal amounts and some patients had very low blood albumin concentrations. This again indicates the varying degrees of systemic inflammation present in our patient population.

Average LPC was in the range of 200–300 μM, which could be considered as the normal range from the few existing studies which included healthy volunteers as well as patients (see Introduction). As reported previously, LPC concentrations were found to be diminished in the blood of patients with leukemia, malignant lymphoma as well as renal and gastrointestinal tumours. But within our group the examined LPC concentrations did not differ significantly between the sexes or among the tumour diagnoses (see Table 3). Therefore, the attenuation of LPC concentrations might occur independent of the type of malignant disease.

**Table 3:** Average LPC concentrations classified by sex and tumour diagnosis with calculation of statistically significant differences between means

	**subgroup**	**n**	**average LPC conc. (μM)**	**standard deviation (μM)**	**range (μM)**	significant difference*
**sex**	male	26	202	53	89–305	no
	female	33	212	64	109–362	no

**cancer type**	breast	17	205	60	128–239	
	prostate	6	198	47	122–249	
	lung	5	177	38	118–214	no
	lymphoma	8	227	80	98–356	
	gastrointestinal	12	194	53	89–270	
	other^2^	11	232	60	135–362	

^2 ^ovaries, kidney, bladder, uterus, mesothelioma, myeloma, aggressive fibromatosis, oligodendroglioma

* p < 0.05

### Correlation of LPC with inflammatory and nutritional parameters (see Tables 4&5)

**Table 4:** Correlation between LPC concentrations and the examined parameters (n = 59)

**parameter correlated with LPC**	**correlation coefficient r**	**p**	significant*
**age (years)^1^**	-0.159	0.229	no
**performance status^1^**	-0.12	0.365	no
**BMI (kg/m^2^)**	0.0097	0.9416	no
**FM (% BW)**	0.2483	0.058	no
**ΔFD (% BW)**	0.3099	0.0169	yes
**HPO (FORT units)^1^**	-0.493	0.0000	yes
**CRP (mg/dl)^1^**	-0.482	0.0000	yes
**ECF (% TBW)**	-0.5531	0.0000	yes
**albumin (g/dl)^1^**	0.454	0.0000	yes

^1^non-normal distribution; correlation tests performed with Spearman rank correlation test

* p < 0.05

**Table 5:** Correlation between LPC levels and the examined parameters in various subgroups

**subgroup**	**Δ FD vs. LPC**	**HPO^1 ^vs. LPC**	**CRP^1 ^vs. LPC**	**ECF vs. LPC**	**albumin^1 ^vs. LPC**
**breast (n = 17)**	r = 0.2646	**-0.811**	-0.473	**-0.666**	0.325
	p = 0.3047	0.0000*	0.0537	0.0035*	0.196
**prostate (n = 6)**	-0.347	-0.257	-0.507	-0.534	0.638
	0.5	0.658	0.297	0.275	0.175
**lung (n = 5)**	-0.291	-0.7	0.000	0.51	-0.7
	0.634	0.233	1.000	0.38	0.233
**lymphoma (n = 8)**	**0.777**	-0.595	-0.655	**-0.731**	0.401
	0.0233*	0.102	0.0716	0.0394*	0.29
**GIT (n = 12)**	0.382	0.055	-0.567	-0.514	**0.653**
	0.22	0.852	0.0512	0.0873	0.0203*
**other (n = 11)**	0.258	-0.482	-0.397	-0.48	**0.693**
	0.443	0.124	0.21	0.135	0.0321*
**Δ FD>10%loss (n = 15)**	**0.5315**	-0.136	**-0.575**	**-0.6612**	0.386
	0.0414*	0.62	0.0241*	0.0153*	0.15
**BMI ≤ 21 (n = 9)**	0.422	**-0.833**	0.008	**-0.828**	0.51
	0.258	0.0019*	0.948	0.0059*	0.138
**CRP > 1 (n = 22)**	0.337	-0.301	**-0.427**	**-0.4691**	0.33
	0.125	0.17	0.0467*	0.0276*	0.131
**HPO ≥ 500 (n = 18)**	0.014	-0.29	**-0.519**	-0.318	0.384
	0.956	0.238	0.0269*	0.198	0.114
**alb. < 3,5 (n = 10)**	0.217	-0.345	**-0.713**	-0.583	0.428
	0.546	0.309	0.0186*	0.0769	0.199

^1^non-normal distribution; correlation tests performed with Spearman rank correlation test

* significant correlation (p < 0.05)

Upper number in cells corresponds to correlation coefficient r of respective pair analysis, lower number corresponds to p (as displayed in first cell)

The range of LPC concentration observed in our group of cancer patients was rather large. This broad distribution justified the investigation of the correlation of LPC with the parameters of inflammation (CPR, HPO) and nutritional parameters (ECF, albumin, weight loss, fat mass, BMI).

### LPC and parameters of weight

LPC showed no significant correlation with the age of the patients examined, therefore the LPC concentration in blood plasma appears to be independent of the age. Neither BMI nor relative fat mass correlated with the LPC concentration. In contrast, LPC correlated with the relative change in body weight as well as with HPO, albumin, CRP and ECF. We found LPC to correlate directly with the weight change since cancer diagnosis considering the whole group of patients (r = 0.3099). Narrowing down to a subgroup of patients with severe weight loss since first diagnosis reveals this interrelation even stronger (r = 0.5315). We conclude that LPC concentrations may not be influenced by body weight itself. Underweight or overweight do not allow any predication of LPC concentrations, but if a change in weight has occurred, the LPC is prone to have changed, too. That means LPC concentration is lowered when a loss of weight – in our patients group caused by the cancer disease – could be observed. In consequence, it might be possible that a low LPC concentration is an indicator for the actual process of losing weight and LPC concentrations might return to normal when the weight stabilises even if the patient has reached underweight.

### LPC and parameters of inflammation

Since disease-related weight loss is known to often be associated with inflammatory processes, our findings regarding markers of inflammation and metabolic activity support the observed relationship between LPC and weight loss. First of all, we found HPO to correlate with LPC in the whole group and even stronger in the group of patients with breast cancer (r = -0.811). Regarding only the patients with a BMI equal to or lower than 21 which is only a small subgroup in our investigation, we also see a strong correlation between HPO and LPC (r = -0.833) leading us to consider whether unduly high metabolic activity that might lead to weight loss in cancer patients is indicated by decreased LPC concentrations, too.

Furthermore, we found CRP to correlate negatively with LPC concentration (r = -0.482). CRP higher than 1 mg/dl is generally considered as status of inflammation. Restricting the patients' group to those who had an elevated CRP concentration in their blood, we observed a slightly weaker correlation between LPC and CRP than in the whole group. This might be explained by the higher mathematical influence here of one single value, 21.1, which is the only one greater than 10. Regarding only patients with elevated HPO concentrations we find a stronger correlation between LPC and CRP as well as in the subgroups of patients with severe weight loss and with low plasma albumin concentrations (r = -0.713). This supports our assumption that parameters of inflammation, weight loss and LPC are clearly interrelated. Just about not significant as defined by general convention (p < 0.05) but still worth considering are the correlations between LPC and CRP in the subgroups of breast cancer patients, lymphoma patients and patients with gastrointestinal cancers.

This leads us to speculate that LPC concentrations are associated with a high metabolic activity and activated inflammatory processes and thus with syndromes typically associated with disease severity. LPC concentrations might thus be a general indicator of disease severity.

### ECF and albumin

Another parameter associated with inflammation and general unfavourable state of health is the extra cellular fluid in body tissues as percentage of total body water. High ECF in relation to intra cellular fluid is often caused by inflammatory processes that augment vascular permeability and lead to local oedema, ascites or pleural effusions [21]. Another cause can be a disturbed osmotic balance between the blood and the tissue compartment. If the osmotic pressure of the blood is lowered, more liquid is pushed out of the vessels into the surrounding tissues [22]. Osmotic pressure in the blood is held up by its content of proteins, 60% thereof being albumin. An albumin deficiency can therefore cause oedema and ascites. Albumin is considered to be an inverse inflammatory marker, decreasing when inflammation is present [23]. We found ECF to show strong inverse correlations with LPC concentrations. Both negatively in the whole group as even stronger in several subgroups like the breast cancer (r = -0.666) and the lymphoma patients. Same can be said for the subgroups of patients with more than 10% weight loss since first diagnosis and patients with low BMI as well as patients with elevated CRP. Patients with high ECF have lowered LPC concentrations. ECF also correlated negatively with albumin concentrations and albumin correlated directly with LPC (r = 0.454). Thus, patients with lowered albumin had diminished LPC amounts in their blood. Since albumin is a regulating factor both for ECF because of the osmotic pressure balance, and for LPC having 2.8 binding sites for this molecule [24], one could conclude that the relationship between LPC and ECF is solely explained by their common regulator albumin. If we only consider patients with albumin between 4 and 5 g/dl (n = 22) we do not observe any correlation between ECF and LPC which would confirm that LPC and ECF only interrelate because of their dependency on albumin.

On the other hand, we do not observe significant correlations between LPC and albumin where we find them between LPC and ECF, for example in the breast cancer group. Therefore, we suppose that the interrelation of LPC and ECF is not exclusively accounted for by albumin.

## Conclusion

For the first time, we provided evidence that there exists a relationship between LPC concentrations in blood plasma and both weight change and inflammatory processes in malignant diseases. Reason for these observations might be the observed rapid PL-turnover of tumour cells resulting in an increased LPC consumption from the blood plasma. Since an increased turnover of membrane PL appears to be associated with tumour progression and metastasis, LPC blood concentrations might well be a strong marker for tumour progression. Since we did not have many patients in our examined group who suffered from severe weight loss or underweight, the observations we made concerning the body weight and its relationship with LPC should be confirmed in a study with a group of patients where weight loss occurs more often and more severe. Further, the effects of a nutritional supplementation with phospholipids on the weight loss of cancer patients should be investigated.

## Methods

### Patients

We studied 59 patients (33 women, 26 men). All participants gave informed written consent. The compliance of study protocol with the Helsinki Declaration was approved by the Ethics Committee of Freiburg University. Patients were recruited as in-patients from the Department of Medical Oncology at Tumor Biology Center Freiburg between November 2005 and February 2006. Inclusion criteria were: age >18 years, diagnosis of solid tumour and performance status <3. Mean age of the patients was 60.6 years, mean body mass index was 25.6 kg/m^2 ^and mean performance status was 1.5 (see Table 2). Patients differed considerably with respect to BMI (and thus nutritional status) and performance status (see Table 2) as was intended to allow valid correlation analyses. All patients had been diagnosed with a solid tumour (see Table 3) and had been receiving varying treatments.

### Blood sampling and analysis

Postabsorptive blood samples were collected in EDTA-containing tubes for routine analysis, plasma was separated by centrifugation and analysed immediately or stored in aliquots at -80°C until LPC analysis. CRP was determined using a sandwich immunoassay (VITROS Chemistry Products CRP Slides; Ortho-Clinical Diagnostics Inc., New York, USA) and albumin using a colorimetric assay (VITROS Chemistry Products ALB Slides; Ortho-Clinical Diagnostics Inc., New York, USA).

Hydrogen peroxides were determined from whole blood by a photometric assay containing Fenton's reagent (FORT test, Micro-Medical Instrumente GmbH, Königstein, Germany). For this assay, 20 μl of capillary blood were taken from the patients' finger, mixed with the reaction solution, centrifuged and transferred to a cuvette for photometric measurement at 505 nm wavelength (FORM-CR 2000, Micro-Medical Instrumente GmbH, Königstein, Germany). Results are expressed as arbitrary FORT units (1 unit corresponding to 7.6 μMol/l hydrogen peroxide). The test is reliable with an intra-assay Vc<5% [18]. The normal range in healthy volunteers has been reported as 230–310 units [17].

### Examination of nutritional status

Participants were weighed and measured and BMI was calculated as (weight in kg)/(height in m)^2^.

Bioelectrical impedance analysis (BIA) estimates body water from whole body impedance against an alternating high-frequency current. For the test a low voltage is applied to wrist and ankle via self-adhering electrodes (Fresenius Kabi AG, Medical Devices, Bad Homburg, Germany) and an alternating current produced of about 0.8 mA [13]. The analysis was performed using a calibrated impedance spectrum analyzer (Hydra 4200, Xitron Technologies Inc., San Diego, USA), an alternating current at multiple frequencies from 5 to 1000 kHz and non-linear regression analysis using the Cole-Cole method. Further parameters obtained are fat free mass (FFM), total body water (TBW) as well as intracellular (ICF) and extracellular water (ECF) [25].

### LPC analysis

In order to analyse LPC in blood plasma, samples had to be subjected to lipid extraction prior to HPTLC analysis.

Extraction was performed according to a modified method of Bligh and Dyer [26] as follows: 75 μl of the serum sample was mixed with 925 μl 0.9% NaCl aqueous solution as well as 500 μl of each of the five calibration standards with 500 μl 0.9% NaCl aqueous solution. Then, 3 times in repetition, 2 ml CHCl_3_/MeOH (2:1 v/v) were added to each vial, vials were shaken for 5 min. at 1000 rpm, then centrifuged at 4°C for 10 min. at 3000 rpm (2060 × g) and the CHCl_3 _layer was transferred to a clean, dry glass vial. The collected CHCl3 phases were evaporated at 40°C under a stream of nitrogen. Dry serum extracts were dissolved in 75 μl, calibration standards in 500 μl of Hexan/Isopropanol/H_2_O (40/50/8 v/v/v) by shaking again for 5 min. at 1000 rpm. HPTLC silica gel plates (Merck, 20 × 10 cm) were preconditioned by a blanc run in the mobile phase same as used for the later analysis: CHCl_3_/MeOH/H_2_O/NH_3 _(65/24/4/0,4 v/v/v/v) and dried on a heating plate at 180°C.

Twelve μl of each calibration standard and of each sample were applied to the preconditioned plate as bands of 8 mm width with the Camag Automatic Sampler TLC III. Each plate was developed with the above mentioned mobile phase in a closed glass tank after letting the solvent equilibrate in the tank. Run time was about 16 min. with 10 cm distance (the whole length of the plate). After development plates were dried on a heating plate at 180°C for 10 min. For detection purposes the plates were stained with a copper sulphate/phosphoric acid solution (14,7% w/v; 10% v/v) by 3 times repeated immersion for 2 sec. with the help of the automatic Camag Immersion device III and then dried for 6 min. at 160°C in the oven of an GC apparatus. After staining, the plates could be interpreted by scanning with a Camag TLC-Scanner II (CATS-Software, version 3.16) with a tungsten bulb at 530 nm in absorption/reflection mode.

Results were calculated from the calibration curve established by the 5 calibration standards, solutions of LPC (Sigma, Germany) in 0.9% NaCl aqueous solution, ranging from 60 to 480 μM in concentration.

### Statistics

The statistical analysis was performed with SigmaStat 3.1 (Systat Software Inc., USA, 2004).

Analysis of differences was performed with Student's t-test. Correlation tests were performed with Pearson's correlation test if the parameters were normally distributed, all others were performed with the Spearman rank correlation test.

## Abbreviations

LPC = lyso-phosphatidylcholine; PC = phosphatidylcholine; BMI = body mass index; ΔFD = weight change since first diagnosis; BW = body weight; CRP = C reactive protein; HPO = hydrogen peroxides; ECF = extracellular fluid; PLA_2 _= phospholipase A_2_; LCAT = lecithin-cholesterin-acyl-tranferase; MF-BIA = multiple frequency bioelectrical impedance analysis; FFM = fat free mass; TBW = total body water; ICF = intracellular fluid; HPTLC = high performance thin layer chromatography; QC = quality control; FOR = free oxygen radicals; LOQ = limit of quantification; LOD = limit of detection

## Competing interests

None of the authors had any financial or personal interest in any company or organization sponsoring the research, including advisory board affiliations.

## Authors' contributions

JA and AKW designed and conducted the patients' study. CU supervised the clinical work. UM designed the experimental setup. LAT performed the experimental work and analyzed the data. LAT and UM wrote the manuscript, with input from all authors. All authors have read and approved the final manuscript.
